# Alu hypomethylation in naturally and surgically postmenopausal women; a cross-sectional study

**DOI:** 10.1371/journal.pone.0273403

**Published:** 2022-08-25

**Authors:** Pattarawadee Siriprapanonkul, Nalina Orprayoon, Punkavee Tuntiviriyapun, Phanupong Phutrakool, Unnop Jaisamrarn, Apiwat Mutirangura, Sukanya Chaikittisilpa

**Affiliations:** 1 Department of Obstetrics and Gynecology, Faculty of Medicine, Chulalongkorn University and King Chulalongkorn Memorial Hospital, Bangkok, Thailand; 2 Menopause Research Group, Department of Obstetrics and Gynecology, Faculty of Medicine, Chulalongkorn University and King Chulalongkorn Memorial Hospital, Bangkok, Thailand; 3 Chula Data Management Center, Faculty of Medicine, Chulalongkorn University, Bangkok, Thailand; 4 Center of Excellence in Molecular Genetics of Cancer and Human Diseases, Department of Anatomy, Faculty of Medicine, Chulalongkorn University, Bangkok, Thailand; Ruđer Bošković Institute, CROATIA

## Abstract

Menopause, which may accelerate the hallmarks of the natural aging process, represents a point in time characterized by the permanent cessation of menstruation following the loss of ovarian estrogen production. Unlike natural menopause, which is characterized by a gradual decrease in estrogen production, when both ovaries are removed before the natural age of menopause, the onset of estrogen deprivation is abrupt. Further, a decrease in genome methylation frequently occurs in aging cells, and the major interspersed repetitive DNA elements in humans are Alu elements. In blood cells, Alu demethylation starts at an age of approximately 40 years, and increases with age. Here, we explored the Alu methylation levels corresponding to age-matched pre-menopausal, naturally postmenopausal, and surgically postmenopausal women aged 45–55 years (n = 60 in each group). Our results indicated that the body mass index (BMI), time-since-menopause, and Alu methylation levels corresponding to the three groups were significantly different. However, no correlations between Alu methylation level and BMI, time-since-menopause, or age were observed. Additionally, the Alu methylation level corresponding to the natural post-menopause group was significantly lower those corresponding to the pre-menopausal (p = 0.001) and surgical post-menopausal (p = 0.037) groups. In conclusion, Alu hypomethylation occurs in naturally postmenopausal women, implying that when women reach the age of natural menopause, the cell aging process may progress significantly with genome hypomethylation. These findings, notwithstanding, further studies are necessary to clarify whether bilateral oophorectomy before the age of menopause affects the cell aging process to a greater extent than natural menopause, and whether estrogen therapy or other interventions can delay cell aging in this regard.

## Introduction

Aging, which is characterized by a progressive loss of physiological integrity, leads to impaired body function and increased risk of disease and death [1]. Reportedly, the life-time risk of developing a non-communicable disease (NCD) at age 45 years and above is 90%. Moreover, it has been observed that at least one-third of NCDs subsequently develop into multiple NCDs [2]. In women, several long-term health effects of aging, including osteoporosis, cardiovascular disease, stroke, and cognitive impairment, increase after menopause, which represents a point in time that is characterized by the permanent cessation of menstruation following the loss of ovarian activity, especially estrogen production. Estrogen plays an important role in the regulatory mechanisms required for tissue maintenance and repair, such as mitochondrial function, intracellular communication, stem cell division, and circadian rhythms [3–7]. The loss of estrogen production may accelerate the hallmarks of the natural aging process, including genomic instability, telomere attrition, epigenetic alterations, loss of proteostasis, deregulated nutrient sensing, mitochondrial dysfunction, cellular senescence, stem cell exhaustion, and altered intercellular communication [1].

Furthermore, epigenetic regulation involves three activities, namely, DNA methylation, chromatin remodeling, and histone protein modification, and it has been observed that global hypomethylation reduces genome methylation at interspersed repetitive sequences (IRSs), which are major contributors to genome size, accounting for approximately 45% of human genomic DNA [8]. Under normal circumstances, IRSs are methylated to varying degrees depending on their location [9]. However, global hypomethylation, primarily owing to the loss of IRS methylation, occurs with the aging process [10], and among IRSs, Alu, which prevents endogenous DNA damage, is the most abundant short intersperse element [8, 11]. Owing to Alu hypomethylation, DNA accumulates the damage and becomes unstable [12]. It has also been reported that aging and aging-related diseases, such as postmenopausal osteoporosis, are associated with Alu hypomethylation [12–14].

In natural menopause, estradiol levels do not gradually reduce during the period before menopause. Rather, they remain within the normal range and even become slightly elevated until approximately 1 year before the cessation of follicular growth and development [15]. Bilateral salpingo-oophorectomy, which is performed before the natural age of menopause and is defined as surgical menopause, is associated with overall mortality and adverse long-term health effects, such as the deterioration in bone health, cardiovascular health, and sexual as well as cognitive function [16]. Thus, different menopausal statuses may not show identical cell aging processes. In this study, we aimed to determine the Alu methylation levels in women with different menopausal statuses to the end of evaluating the cell aging process that occurs in women owing to menopause.

We analyzed the levels and patterns of DNA methylation owing to dissimilar alterations in IRS DNA methylation. Previously, using Combined Bisulfite Restriction Analysis (COBRA), we showed the existence of not only a general, but also a locus-specific influence of long interspersed nuclear element 1 and Alu methylation as well as the associated pattern [17]. Presently, there are various techniques, including pyrosequencing and COBRA that offer the possibility to accurately measure methylation levels [18]. In this study, we used COBRA to evaluate Alu methylation levels. Our results indicated that Alu hypomethylation occurs in naturally postmenopausal women, and that the cell aging process may progress significantly as the genome is hypomethylated when women reach the age of natural menopause.

## Materials and methods

### Subjects

A total of 180 women were enrolled at the Menopause Clinic, King Chulalongkorn Memorial Hospital, Bangkok, Thailand, within the December 2018 to September 2019 period. The participants were assigned to three groups, namely, the pre-menopausal, natural postmenopausal, and surgically postmenopausal groups (n = 60 per group). The subjects were recruited according to the following criteria: 1) provided consent to participate, 2) aged 45–55 years, and 3) premenopausal women who had regular menstrual cycle with serum follicle-stimulating hormone levels < 25 IU/ml, or naturally postmenopausal women who had a history of 12-month amenorrhea with serum follicle-stimulating hormone levels > 40 IU/ml. Further, the time-since-menopause was 1–10 years, or surgically postmenopausal women who had undergone bilateral oophorectomy before menopause. Women with a history of cancer, diabetes mellitus, hypertension, osteoporosis, autoimmune diseases, smoking, history of chemotherapy, history of radiation therapy, or those who were administered hormone therapy within the past six months were excluded. Demographic data, including age, menopausal status, time-since-menopause, weight, height, and body mass index (BMI), were collected. The main outcome was Alu methylation level (%) in peripheral blood cells.

First, we conducted a pilot study to measure the Alu methylation levels in blood samples from premenopausal, naturally postmenopausal, and surgically postmenopausal women. This preliminary study showed Alu methylation levels of 75.07 ± 0.40%, 72.55 ± 0.60%, and 73.72 ± 0.01%, for the three groups, respectively. Thereafter, the sample size was calculated based on the pilot study data using the formula for each specific population (infinite population mean) with standard deviation = 0.6, error = 0.2, and alpha = 0.01. The final sample size was 60 participants per group, with age-matched controls.

### Blood sample collection and DNA preparation for COBRA Alu

Heparinized whole blood samples (3 ml) were centrifuged to remove the buffy coat containing white blood cells. After the addition of 1 ml of red blood cell (RBC) lysis buffer into the samples, centrifugation was performed at 1,700 RCF for 3 min and the supernatant was discarded [13]. This step was repeated until the RBCs were completely lysed. Next, 950 μl of RBC lysis buffer-II, 50 μl of 10% sodium dodecyl sulfate, and 20 mg/ml of proteinase kinase were added and the resulting solution was incubated overnight at 50°C until the cells were digested. Thereafter, one volume of a solution containing phenol, chloroform, and isopropanol in the ratio 25:24:1 was added followed by centrifugation at 14,000 rpm for 5 min. DNA was then separated and added to a solution containing 500 μl of 100% ethanol and 500 μl of 10 M ammonium acetate, followed by centrifugation at 14,000 rpm for 15 min. The resulting supernatant was discarded, and subsequently, 500 μl of 70% ethanol was added, followed by centrifugation at 14,000 rpm for 5 min. Finally, the supernatant was discarded, while the DNA sediment was air dried for 1 h, and subsequently diluted with 20 μl of distilled H_2_O and incubated at 37°C for 10 min. Then, the DNA quantity was measured using a NanoDrop 2000 device (Thermo Fisher Scientific, Waltham, MA, USA).

### COBRA

Alu methylation levels were measured using the ALU-COBRA method. In brief, DNA extraction was performed using the standard phenol chloroform extraction method. All the DNA samples were treated with sodium bisulfite essentially following the manufacturer’s instructions (EZ DNA Methylation-Gold Kit; Zymo Research, Irvine, CA, USA). Then, to investigate Alu methylation, the bisulfite-treated DNA samples were subjected to 45 cycles of PCR with Alu-Forward (5′-GGYGYGGTGGTTTAYG TTTGTAA-3′) and Alu-Reverse (5′-CTAACTTTTTATATTTTTAATAAAAACRAAATTTCACC A-3′) primer sequences to generate a product that was 133 bp long [19]. After the PCR amplification process, the Alu amplicons (133 bp long) were digested with TaqI in TaqI buffer (MBI Fermentas, Burlington, Canada); the digestion reaction mixture was incubated at 65°C for 16 h. Thereafter, the Alu element digested products were electrophoresed on an 8% non-denaturing polyacrylamide gel and stained with SYBR green nucleic acid gel (Gelstar, Lonza, Rockland, ME, USA). The band intensity of COBRA Alu methylation was then measured using Azure™ Biosystems c300 (Azure Biosystems, Dublin, CA, USA), with distilled water as the negative control. All the experiments were performed in duplicates, and DNA samples from HeLa, Jurkat, and Daudi cell lines, from the same cell pool and passage, were used as positive controls in every experiment to standardize inter-assay variations. Notably, the HeLa band in each gel band was measured, and thereafter, calculations were made to adjust the results corresponding to each gel. The methylation levels are reported as the percentage of methylated CpG [20].

### Statistical analysis

Statistical analysis was performed using STATA software version 15 (Stata Corp LLC., College Station, TX, USA). Demographic data were presented as percentages and medians with interquartile ranges, and the Kruskal–Wallis test followed by Dunn’s test were performed to compare differences between the menopausal status groups. Further, the degree of the correlations between Alu methylation levels and the associated covariates were assessed via Spearman’s correlation analysis, and box and whisker plots were generated using GraphPad Prism version 9.0.0 for Windows (GraphPad Software, San Diego, CA, USA). Statistical significance was set at *P* < 0.05.

### Ethics statement

This cross-sectional, age-matched study was conducted in accordance with the Declaration of Helsinki, and all the procedures involving human subjects were approved by the Institutional Review Board of the Faculty of Medicine, Chulalongkorn University (Approval number 230/61). All the study participants provided written informed consent.

## Results

In total, 180 women were recruited in this study. The three groups included: premenopausal women, naturally postmenopausal women, and surgically postmenopausal women. Further, each group consisted of 60 age-matched participants, and the average age of all these participants, all of whom were non-smokers, was 50.7 ± 2.3 years. Table 1 provides details regarding the demographic data corresponding to the participants in each group. A Kruskal–Wallis test was performed to determine if the associated variables differed among the three groups. Thus, we observed significant differences among the three groups with respect to BMI, time-since-menopause, and Alu methylation level.

**Table 1:** Demographic data corresponding to the participants in each group and Post-hoc comparison.

Variables	Total	Overall (n = 180)	Menopause status, Median (IQR)			*H*	*P* value	*Post-hoc comparison*		
			Pre (n = 60)	Natural (n = 60)	Surgical (n = 60)			1^st^ group (Pre vs. Natural)	2^nd^ group (Pre vs. Surgical)	3^rd^ group (Natural vs. Surgical)
BMI	180	24.4 (21.6–27.6)	24.9 (22.5–28.3)	23.2 (21.2–24.9)	26.2 (21.8–28.3)	8.988	0.011^a^	0.022^a^	> 0.999	0.010^a^
Year since menopause, year	120	3 (2–5)	-	3 (1–4)	4 (2–6)	6.287	0.012^a^			
Total Alu level (%)	180	58.9 (56.6–61.6)	60.0 (58.1–62.6)	57.6 (55.6–60.0)	59.6 (55.5–62.1)	12.633	0.002^b^	0.001^b^	0.308	0.037^a^
Smoking, n (%)	180					-	-			
• No		180 (100)	60 (100)	60 (100)	60 (100)					
• Yes		-	-	-	-					

^a^Significance level at 0.05 (two-tailed)

^b^Significance level at 0.01 (two-tailed)

BMI, body mass index; IQR, interquartile range; Pre, pre-menopausal women; natural, naturally postmenopausal women; surgical, surgically postmenopausal women.

**Fig 1:**
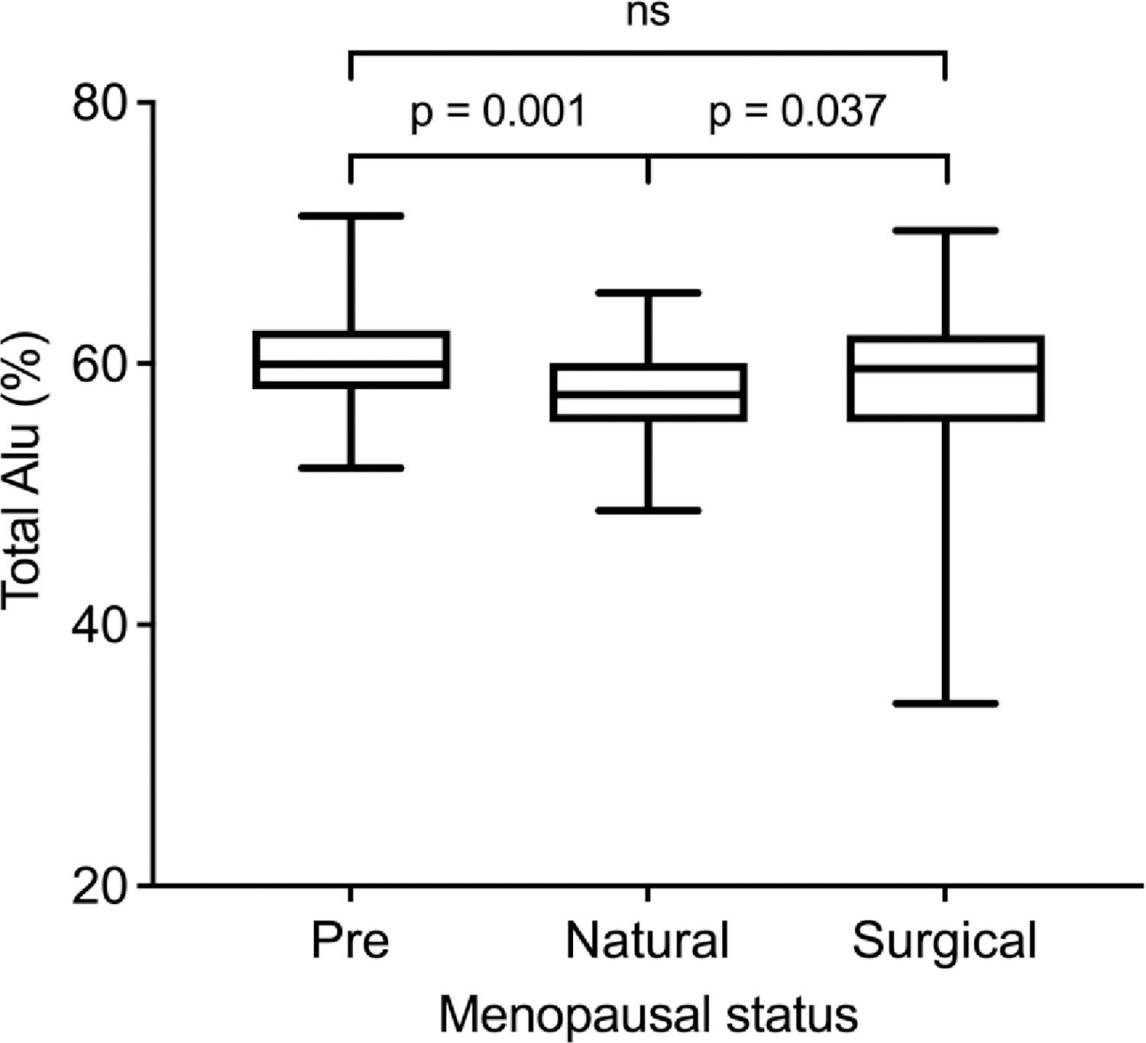
Variation in the total Alu level (%) with menopausal status. Dunn’s post-hoc test showing differences in the total Alu level. Statistical significance was set at p < 0.05.

Dunn’s post-hoc test was performed to determine whether menopausal status was associated with changes in BMI and Alu methylation levels. In this regard, we observed that BMI was significantly different in the 1st group (pre-menopause vs. natural; *P* = 0.022) and the 3rd group (natural vs. surgical; *P* = 0.010). Further, the Alu methylation levels were significantly different in the 1st group (pre-menopause vs. natural; *P* = 0.001 and the 3rd group (natural vs. surgical; *P* = 0.037) (Table 1 and Fig 1).

**Fig 2:**
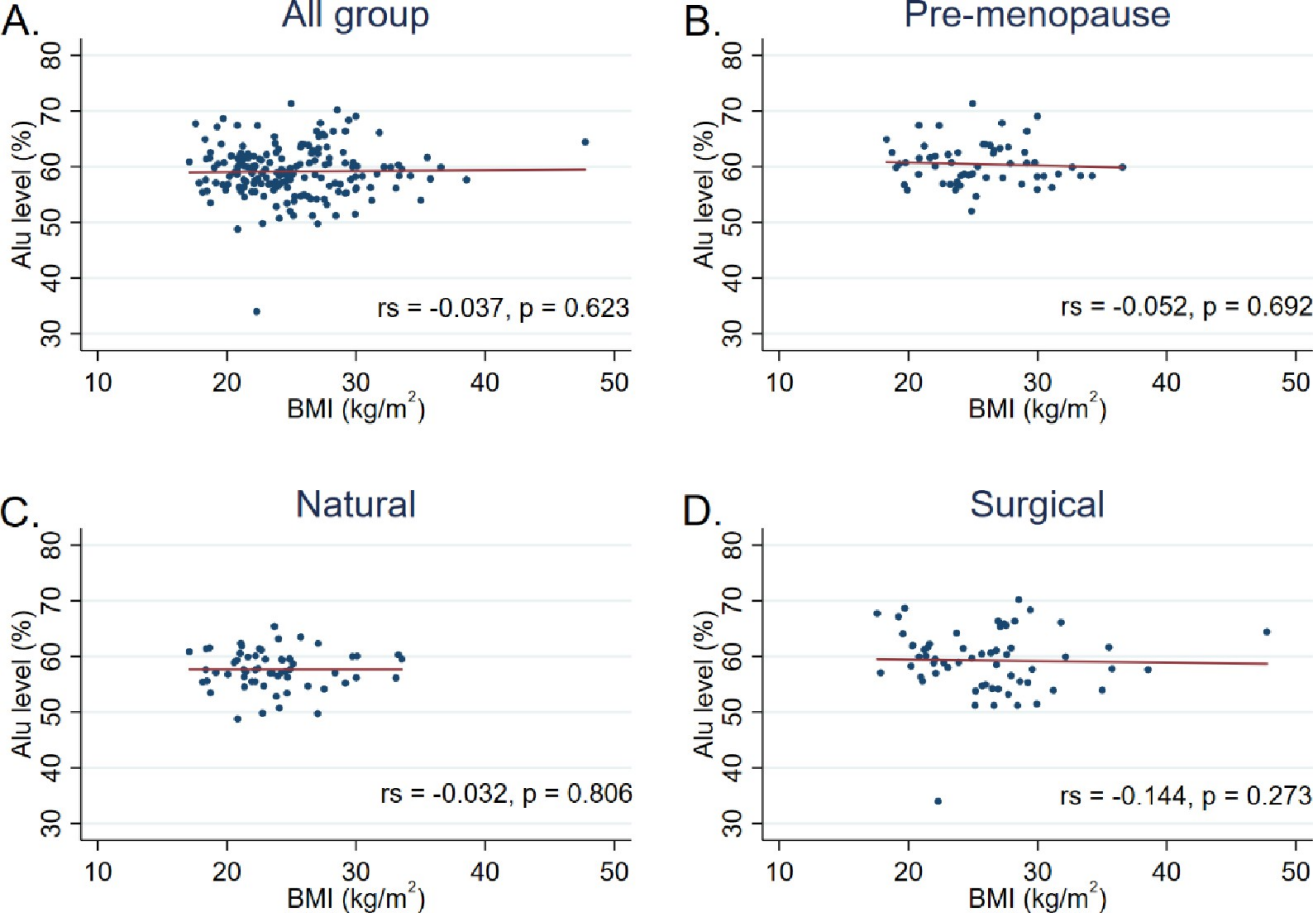
Correlation between the Alu methylation level and BMI among A) all participants, B) pre-menopause group, C) natural menopause group, and D) surgical menopause group. Data were analyzed using Spearman’s correlation analysis.

**Fig 3:**
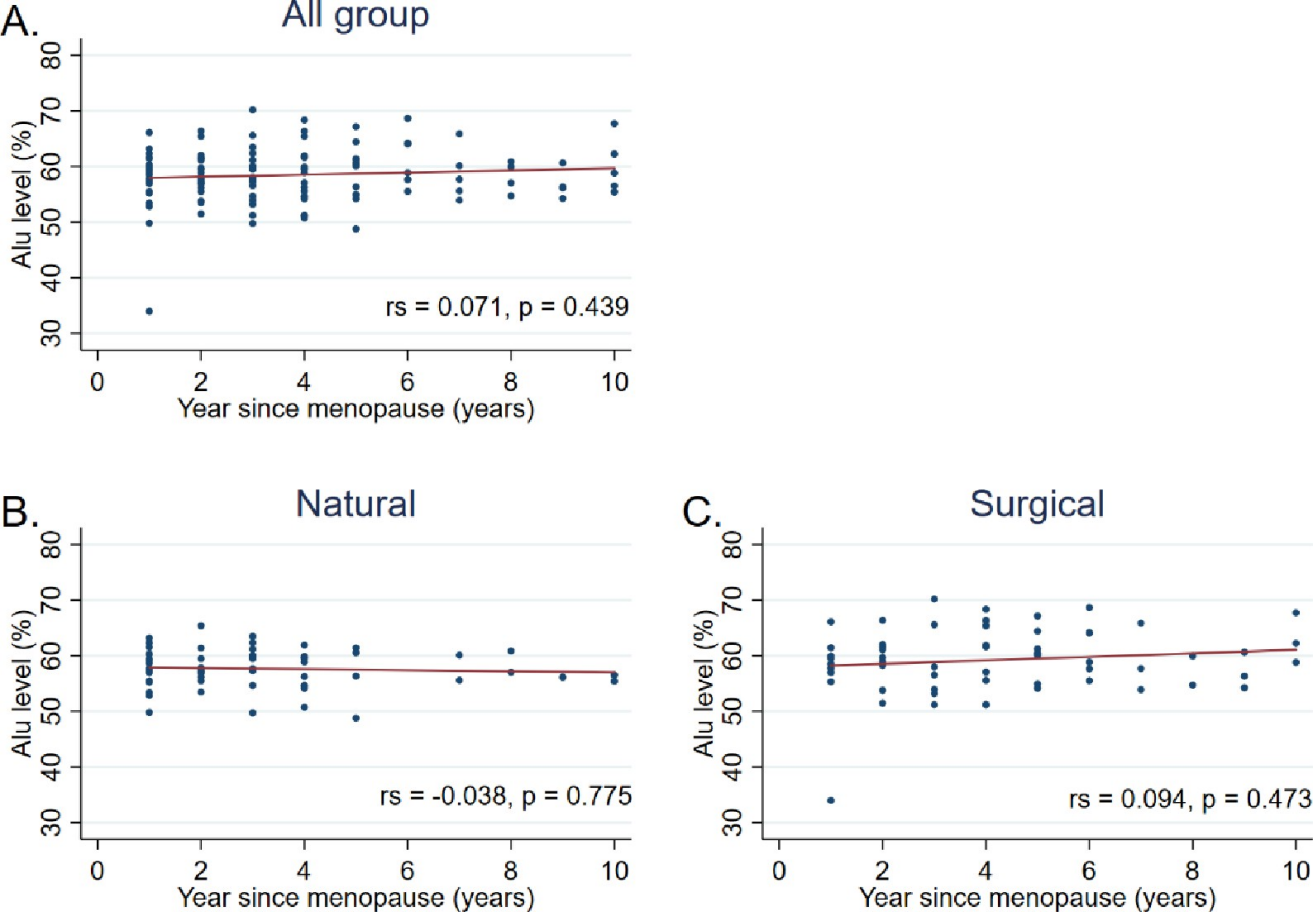
Correlation between the Alu methylation level and time-since-menopause among A) all participants, B) natural menopause group, and C) surgical menopause group. Data were analyzed using Spearman’s correlation analysis.

**Fig 4:**
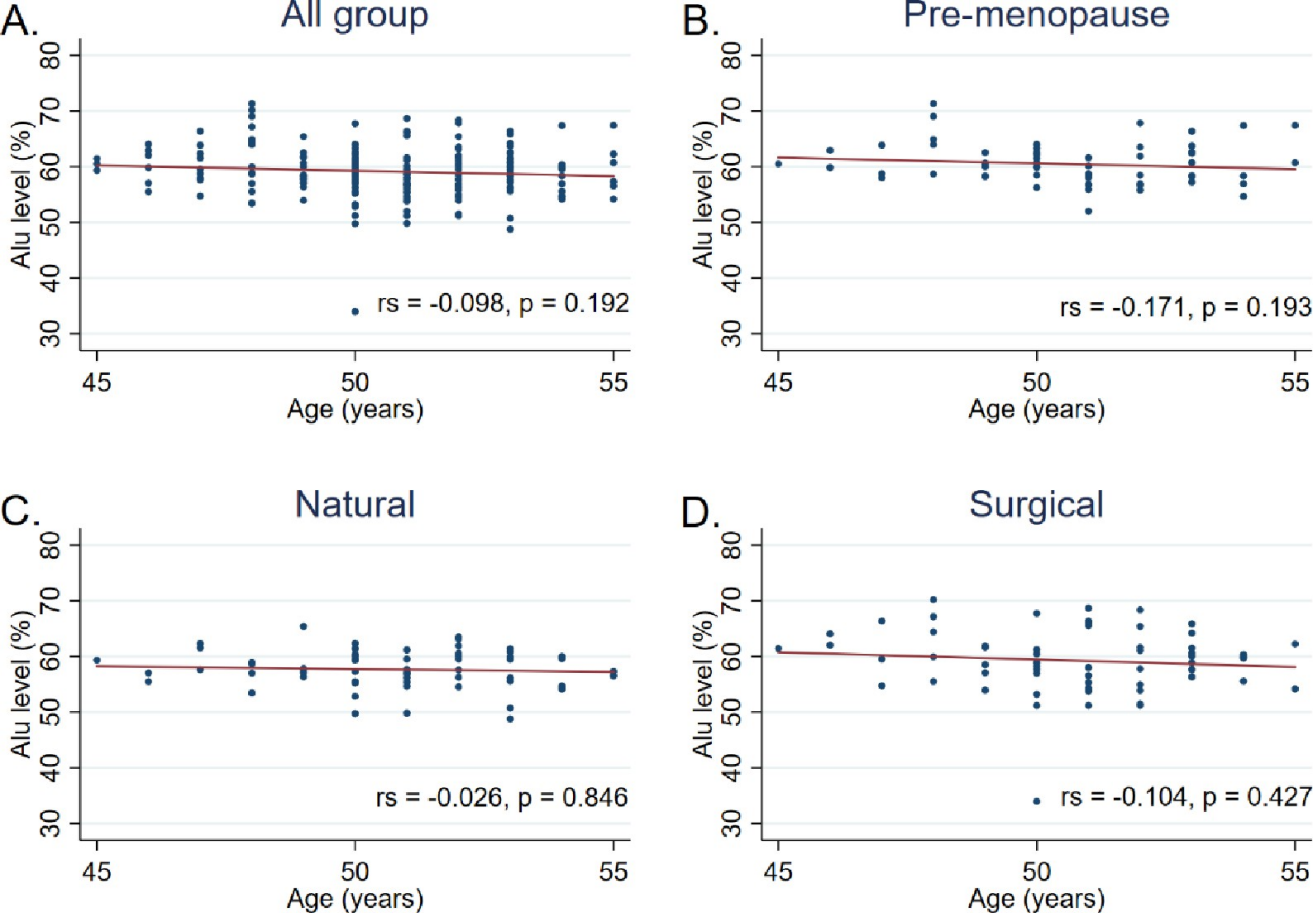
Correlation between the Alu methylation level and age among A) all participants, B) pre-menopause group, C) natural menopause group, and D) surgical menopause group. Data were analyzed using Spearman’s correlation analysis.

Additionally, Spearman’s correlation analysis showed no significant correlation between the Alu methylation level and BMI (*r*_*s*_ = -0037, *P* = 0.623), time-since-menopause (*r*_*s*_ = 0.071, *P* = 0.439), or Age (*r*_*s*_ = -0.098, *P* = 0.192) (Figs 2–4).

## Discussion

The main characteristic of the aging process is genomic instability [1]. In a previous study, it was demonstrated that Alu element methylation is inversely associated with DNA damage in white blood cells [12]. Thus, Alu element methylation may prevent the accumulation of DNA damage and stabilize the genome [20]. However, Alu hypomethylation, which is an age-dependent mechanism, is characterized by IRSs losing methylation at a certain age [21]. Therefore, Alu hypomethylation is a marker that associated with the cell aging process [20].

In this study, we investigated Alu hypomethylation in postmenopausal women and observed that the timing of menopause onset was associated with susceptibility to age-related morbidity and mortality outcomes. After menopause, ovarian production of estrogen decreases, and this diminished availability of estrogen at the cellular and molecular levels may facilitate this aging process [7]. DNA methylation-based patterns have been studied as epigenetic predictors of age or the "epigenetic clock", which is significantly correlated with age and contributes to age-related diseases [22–26]. Specifically, Levine et al. (2016), using the epigenetic clock, demonstrated that menopause may be an epigenetic age acceleration process, i.e., the age at which menopause occurs is associated with biological aging [27]. Our study also demonstrated that Alu hypomethylation, which reportedly, is associated with aging, occurs in naturally postmenopausal women. This may be attributed to the fact that when women reach the age of natural menopause, the cell aging process progresses considerably as the genome of the elderly is hypomethylated [20]. However, the aging process itself may be the cause of natural menopause in women.

The cut-off threshold for the Alu methylation level has been not yet established. The mean Alu methylation level obtained in this study was higher than those reported in previous studies involving postmenopausal women [13]. This higher level of Alu methylation observed in our study group can be attributed to the fact that women with known conditions that could alter the Alu methylation level were excluded, and all our participants, even those in the menopausal groups, could be considered, as otherwise, healthy women.

Ovarian removal before menopause is associated with overall mortality, cognitive decline, loss of bone mass, and increased fracture risk [16]. Although a previous study showed that surgical menopause is associated with an increased epigenetic age [27], our study showed that the Alu methylation level in the surgical menopause group was higher than that in the naturally postmenopausal group. The mechanism in this regard is still unclear, but this observation could be explained by the short period of menopause given that in most of the women, the postmenopausal period was less than 6 years, which is not a long-enough period to demonstrate significant aging changes and the development of common NCDs as reported in a previous study on the effects of premenopausal bilateral ovarian removal [28]. However, our study showed the existence of a trend of Alu hypomethylation in surgically postmenopausal women compared with premenopausal women.

Although the percent differences in Alu levels were small, there were approximately 1 million copies of Alu elements in the human genome and a 1% change in Alu methylation represented a 10,000 loci change in methylation. In a previous study, our research group observed that an increase in Alu methylation by 5% can reduce DNA damage by 75% [12, 14]. Therefore, the removal of both ovaries during hysterectomy solely for ovarian cancer prevention in low-risk women should be cautiously considered as this may trigger the acceleration of the aging process.

To the best of our knowledge, our study may be the first in which Alu methylation in age-matched naturally postmenopausal and surgically postmenopausal women is investigated. Further, our participants were in the early postmenopausal period, which offers the possibility to delay the aging process based on the availability of appropriate interventions. The limitations of the study include the fact that this was a cross-sectional study with convenience sampling. Thus, the results may not be representative of the general population of postmenopausal women. Even though we excluded women with conditions that affect Alu methylation levels, we did not collect data on behavioral factors, including diet and physical activities, which may also influence Alu methylation levels.

## Conclusions

This study showed the occurrence of Alu hypomethylation in naturally early postmenopausal women, which was not the case in surgically early postmenopausal women. Thus, natural menopause may be part of the cell aging process. This notwithstanding, further studies are necessary to determine whether bilateral oophorectomy before the age of menopause can affect the cell aging process, and whether estrogen therapy or other interventions can delay this cell aging.

## Supporting information

S1 File Minimal data set supporting the findings of this study. (XLSX)

S1 Checklist STROBE statement—checklist of items that should be included in reports of *cross-sectional studies*. (DOCX)

## References

[1] Lopez-Otin C, Blasco MA, Partridge L, Serrano M, Kroemer G. The hallmarks of aging. Cell. 2013;153: 1194–1217. doi: 10.1016/j.cell.2013.05.039 23746838 PMC3836174

[2] Licher S, Heshmatollah A, van der Willik KD, Stricker BHC, Ruiter R, de Roos EW, et al. Lifetime risk and multimorbidity of non-communicable diseases and disease-free life expectancy in the general population: A population-based cohort study. PLoS Med. 2019;16: e1002741. doi: 10.1371/journal.pmed.1002741 30716101 PMC6361416

[3] Klinge CM. Estrogenic control of mitochondrial function and biogenesis. J Cell Biochem. 2008;105: 1342–1351. doi: 10.1002/jcb.21936 18846505 PMC2593138

[4] Carruba G, Webber MM, Quader ST, Amoroso M, Cocciadiferro L, Saladino F, et al. Regulation of cell-to-cell communication in non-tumorigenic and malignant human prostate epithelial cells. Prostate. 2002;50: 73–82. doi: 10.1002/pros.10034 11816015

[5] Nakada D, Oguro H, Levi BP, Ryan N, Kitano A, Saitoh Y, et al. Oestrogen increases haematopoietic stem-cell self-renewal in females and during pregnancy. Nature. 2014;505: 555–558. doi: 10.1038/nature12932 24451543 PMC4015622

[6] Brinton RD, Yao J, Yin F, Mack WJ, Cadenas E. Perimenopause as a neurological transition state. Nat Rev Endocrinol. 2015;11: 393–405. doi: 10.1038/nrendo.2015.82 26007613 PMC9934205

[7] Sehl ME, Ganz PA. Potential mechanisms of age acceleration caused by estrogen deprivation: Do endocrine therapies carry the same risks? JNCI Cancer Spectr. 2018;2: pky035. doi: 10.1093/jncics/pky035 31360862 PMC6649786

[8] Batzer MA, Deininger PL. Alu repeats and human genomic diversity. Nat Rev Genet. 2002;3: 370–379. doi: 10.1038/nrg798 11988762

[9] Phokaew C, Kowudtitham S, Subbalekha K, Shuangshoti S, Mutirangura A. LINE-1 methylation patterns of different loci in normal and cancerous cells. Nucleic Acids Res. 2008;36: 5704–5712. doi: 10.1093/nar/gkn571 18776216 PMC2553567

[10] Bollati V, Schwartz J, Wright R, Litonjua A, Tarantini L, Suh H, et al. Decline in genomic DNA methylation through aging in a cohort of elderly subjects. Mech Ageing Dev. 2009;130: 234–239. doi: 10.1016/j.mad.2008.12.003 19150625 PMC2956267

[11] Deininger PL, Moran JV, Batzer MA, Kazazian HH Jr., Mobile elements and mammalian genome evolution. Curr Opin Genet Dev. 2003;13: 651–658. doi: 10.1016/j.gde.2003.10.013 14638329

[12] Patchsung M, Settayanon S, Pongpanich M, Mutirangura D, Jintarith P, Mutirangura A. Alu siRNA to increase Alu element methylation and prevent DNA damage. Epigenomics. 2018;10: 175–185. doi: 10.2217/epi-2017-0096 29336607

[13] Jintaridth P, Tungtrongchitr R, Preutthipan S, Mutirangura A. Hypomethylation of Alu elements in postmenopausal women with osteoporosis. Plos One. 2013;8: e70386. doi: 10.1371/journal.pone.0070386 23990903 PMC3749148

[14] Mutirangura A. Is global hypomethylation a nidus for molecular pathogenesis of age-related noncommunicable diseases? Epigenomics. 2019;11: 577–579. doi: 10.2217/epi-2019-0064 31070049

[15] Taylor HS, Pal L, Seli E. Menopause transition and menopause hormone therapy. Speroff’s clinical gynecologic endocrinology and infertility. 9th ed. Connecticut: Wolters Kluwer; 2020. pp. 580–751.

[16] Adelman MR, Sharp HT. Ovarian conservation vs removal at the time of benign hysterectomy. Am J Obstet Gynecol. 2018;218: 269–279. doi: 10.1016/j.ajog.2017.07.037 28784419

[17] Rerkasem K, Rattanatanyong P, Rerkasem A, Wongthanee A, Rungruengthanakit K, Mangklabruks A, et al. Higher Alu methylation levels in catch-up growth in twenty-year-old offsprings. PLoS One. 2015;10: e0120032. doi: 10.1371/journal.pone.0120032 25807557 PMC4373937

[18] Pobsook T, Subbalekha K, Sannikorn P, Mutirangura A. Improved measurement of LINE-1 sequence methylation for cancer detection. Clin Chim Acta. 2011;412: 314–321. doi: 10.1016/j.cca.2010.10.030 21078311

[19] Thongsroy J, Patchsung M, Mutirangura A. The association between Alu hypomethylation and severity of type 2 diabetes mellitus. Clin Epigenetics. 2017;9: 93. doi: 10.1186/s13148-017-0395-6 28883893 PMC5580285

[20] Jintaridth P, Mutirangura A. Distinctive pattern of age-dependent hypomethylation in interspersed repetitive sequences. Physiol Genomics. 2010;41: 194–200. doi: 10.1152/physiolgenomics.00146.2009 20145203

[21] Bocklandt S, Lin W, Sehl ME, Sa´nchez FJ, Sinsheimer JS, Horvath S, et al. Epigenetic predictor of age. Plos One. 2011;6: e14821. doi: 10.1371/journal.pone.0014821 21731603 PMC3120753

[22] Weidner CI, Lin Q, Koch CM, Eisele L, Beier F, Ziegler P, et al. Aging of blood can be tracked by DNA methylation changes at just three CpG sites. Genome Biol. 2014;15: R24. doi: 10.1186/gb-2014-15-2-r24 24490752 PMC4053864

[23] Marioni RE, Shah S, McRae AF, Ritchie SJ, Muniz-Terrera G, Harris SE, et al. The epigenetic clock is correlated with physical and cognitive fitness in the Lothian Birth Cohort 1936. Int J Epidemiol. 2015;44: 1388–1396. doi: 10.1093/ije/dyu277 25617346 PMC4588858

[24] Marioni RE, Shah S, McRae AF, Chen BH, Colicino E, Harris SE, et al. DNA methylation age of blood predicts all-cause mortality in later life. Genome Biol. 2015;16: 25. doi: 10.1186/s13059-015-0584-6 25633388 PMC4350614

[25] Chen BH, Marioni RE, Colicino E, Peters MJ, Ward-Caviness CK, Tsai PC, et al. DNA methylation-based measures of biological age: Meta-analysis predicting time to death. Aging (Albany NY). 2016;8: 1844–1865. doi: 10.18632/aging.101020 27690265 PMC5076441

[26] Horvath S. DNA methylation age of human tissues and cell types. Genome Biol. 2013;14: R115. doi: 10.1186/gb-2013-14-10-r115 24138928 PMC4015143

[27] Levine ME, Lu AT, Chen BH, Hernandez DG, Singleton AB, Ferrucci L, et al. Menopause accelerates biological aging. Proc Natl Acad Sci USA. 2016;113: 9327–9332. doi: 10.1073/pnas.1604558113 27457926 PMC4995944

[28] Mytton J, Evison F, Chilton PJ, Lilford RJ. Removal of all ovarian tissue versus conserving ovarian tissue at time of hysterectomy in premenopausal patients with benign disease: Study using routine data and data linkage. BMJ. 2017;356: j372. doi: 10.1136/bmj.j372 28167486 PMC5421461

